# On-Site Detection of Aflatoxin B1 in Grains by a Palm-Sized Surface Plasmon Resonance Sensor

**DOI:** 10.3390/s18020598

**Published:** 2018-02-15

**Authors:** Jeong Moon, Jihyun Byun, Hongki Kim, Eun-Kyung Lim, Jinyoung Jeong, Juyuen Jung, Taejoon Kang

**Affiliations:** 1 Hazards Monitoring Bionano Researh Center, Korea Research Institute of Bioscience and Biotechnology (KRIBB), Daejeon 34141, Korea; jmoon@kribb.re.kr (J.M.); jihyunbyun@kribb.re.kr (J.B.); hongkikim@kribb.re.kr (H.K.); eklim1112@kribb.re.kr (E.-K.L.); jjung@kribb.re.kr (J.J.); jyjeong@kribb.re.kr (J.J.); 2 Department of Nanobiotechnology, KRIBB School of Biotechnology, University of Science and Technology (UST), Daejeon 34113, Korea; 3 BioNano Health Guard Research Center, Korea Research Institute of Bioscience and Biotechnology (KRIBB), Daejeon 34141, Korea

**Keywords:** aflatoxin B1, portable SPR, competitive immunoassay, sensor, on-site detection

## Abstract

Aflatoxins (AFs) are highly toxic compounds that can cause both acute and chronic toxicity in humans. Aflatoxin B1 (AFB1) is considered the most toxic of AFs. Therefore, the rapid and on-site detection of AFB1 is critical for food safety management. Here, we report the on-site detection of AFB1 in grains by a portable surface plasmon resonance (SPR) sensor. For the detection of AFB1, the surface of an SPR Au chip was sequentially modified by cysteine-protein G, AFB1 antibody, and bovine serum albumin (BSA). Then, the sample solution and AFB1-BSA conjugate were flowed onto the Au chip in serial order. In the absence of AFB1, the SPR response greatly increased due to the binding of AFB1-BSA on the Au chip. In the presence of AFB1, the SPR response showed little change because the small AFB1 molecule binds on the Au chip instead of the large AFB1-BSA molecule. By using this portable SPR-based competitive immunoassay, the sensor showed low limits of detection (2.51 ppb) and quantification (16.32 ppb). Furthermore, we successfully detected AFB1 in rice, peanut, and almond samples, which suggests that the proposed sensing method can potentially be applied to the on-site monitoring of mycotoxins in food.

## 1. Introduction

Aflatoxins (AFs) are a group of highly toxic and carcinogenic secondary metabolites produced by the fungi *Aspergillus flavus* and *Aspergillus parasiticus* [1]. AFs are found in a wide range of foods and feeds, such as grains, nuts, dried fruits, and spices, under high temperature and humidity [2]. There are four main types of AF: aflatoxin B1 (AFB1), B2, G1, and G2. Among them, AFB1 is known to be the most common toxin and potent cancer-causing factor, which can induce genetic mutation and hepatocellular carcinoma [3,4]. AFB1 is extremely heat stable and thus difficult to remove once produced [5]. In addition, exposure to AFB1 has increased due to climate change and expanding food trade between nations [6,7]. Therefore, many countries have set legal limits for AFB1 ranging from 0 to 50 ppb (parts per billion) to monitor and regulate the level of AFB1 in foodstuffs [8,9]. Several techniques have been employed for the identification of AFs in food, such as high-performance liquid chromatography [10], thin-layer chromatography [11], overpressured-layer chromatography [12], and enzyme-linked immunosorbent assay [13]. Although these methods can detect AFs efficiently, sophisticated equipment or trained personnel are required. Recently, various immuno-sensors have been developed to detect AFs rapidly and routinely. For example, Spinella et al. reported a piezoelectric quartz-crystal-microbalance sensor for the detection of AFB1 in the range of 0.5–10 ppb [14]. Adányi et al. developed an optical waveguide light-mode spectroscopy method for the detection of AFB1 and ochratoxin A, and they identified AFB1 over a range of 0.5–10 ppb using competitive detection and 5–10 ppb using non-competitive detection [15]. In addition, an electrochemical immuno-sensor based on graphene/conducting polymer/Au nanoparticles/the ionic liquid composite film was developed for the detection of AFB1, displaying a dynamic range from 3.2 fM to 0.32 pM [16]. Masinde et al. applied an immune-chromatographic assay to detect AFs in corn and rice, and showed a 0.1 ppb detection limit [17]. Although these techniques showed sensitive AF detection, they still require heavy instrumental systems and complicated fabrication steps, limiting for on-site monitoring of AFs.

Surface plasmon resonance (SPR) is a well-known technology that enables the label-free, real-time monitoring of antibody–antigen interactions [18]. The SPR technique has been widely used to recognize nucleic acids, proteins, peptides, cells, toxins, etc. [19,20]. Since traditional SPR systems are large and heavy, they have limited use for the on-site detection of analytes. Therefore, the miniaturization of SPR systems is important for practical applications of SPR. Previously, we developed a palm-sized SPR sensor in which the beam from a diode laser is modulated by a rotating mirror [21]. This portable SPR system enables us to widen the practicality of SPR technology. In this regard, we applied the portable SPR sensor for the detection of AFB1 in grains. Miniaturized SPR sensors have been developed and applied to detect chemical and biological species by several research groups [22]. In the case of AFB1, however, this is the first work showing how to detect AFB1 with the portable SPR sensor. For the sensitive detection of AFB1, we adopted a competitive immunoassay. Briefly, the surface of an SPR Au chip was sequentially modified by cysteine-protein G and AFB1 antibody, and then treated with bovine serum albumin (BSA) to prevent nonspecific binding. Next, we flowed the sample solution and AFB1-BSA onto the Au chip and observed the SPR response change. When the sample includes AFB1, the anti-AFB1 antibody binds to AFB1. When the sample has no AFB1, the antibody binds to AFB1-BSA instead of AFB1. Since the SPR response is related to the mass change on the Au chip [23], little change in the SPR response occurs when AFB1 binds to the antibody, while a large change is produced when AFB1-BSA binds to the antibody. By comparing the difference in the signal of the two SPR channels, we could quantitatively detect AFB1 in the dynamic range of 16~200 ppb. More importantly, this method allows for the detection of AFB1 in rice, peanut, and almond samples, indicating its potential applicability to the on-site monitoring of AFB1.

## 2. Materials and Methods

### 2.1. Materials

Standards of AFB1 (A6636), zearalenone (34126), AFB1-BSA conjugate (A6655), and polyclonal anti-AFB1 antibody (A8679) were purchased from Sigma-Aldrich (St. Louis, MO, USA). Cysteine-protein G and Au-coated chips were purchased from MiCoBioMed Co., Ltd. (Daejeon, Korea). Phosphate buffered saline (PBS, 10010023) was purchased from Thermo Fisher (Waltham, MA, USA). Methanol (M0583) was purchased from Samchun Chemical (Seoul, Korea). Rice, peanut, and almond were purchased from a local market. Syringe filters (TR-200506) were purchased from Teknokroma (Barcelona, Spain).

### 2.2. Preparation of AFB1-Spiked Food Samples

Five-gram samples of rice, peanut, and almond obtained from the local market were spiked with known concentrations of AFB1 and incubated for 20 min in the fume hoods. The AFB1-spiked samples were extracted with 25 mL of methanol/water (70:30, v/v), and this mixture was shaken for 10 min. The mixture was then filtered with a 0.45 μm syringe filter, and the extracts were used for analysis.

### 2.3. Preparation of the Au Chip and Detection of AFB1 by the Portable SPR Sensor

The Au chip was cleaned with a piranha solution (70% (v/v) H_2_SO_4_ and 30% (v/v) H_2_O_2_) at 60 °C for 10 min and thoroughly rinsed with ethanol and deionized water. This pre-cleaned Au chip was rinsed with PBS (pH 7.4), which was used as the running buffer. The cysteine-protein G (0.1 mg/mL) 100 μL was injected into the SPR channel at a flow rate of 10 μL/min. Then, 100 μL of polyclonal anti-AFB1 antibody (0.1 mg/mL) were flowed into the channel at the same flow rate. Continually, 100 μL of BSA (0.1 mg/mL) were applied to prevent nonspecific binding. Next, 100 μL of the AFB1 sample were introduced into the channel, followed by a 100 μL injection of AFB1-BSA conjugates (1 mg/mL). During the entire experimental procedure, the SPR response was monitored in real time.

### 2.4. Instrumentation

The portable SPR instrument was purchased from MiCoBioMed Co., Ltd. (Daejeon, Korea). The syringe pump (NE-1000) was purchased from New Era Pump Systems Inc. (New York, NY, USA).

## 3. Results and Discussion

### 3.1. Detection of AFB1 by the Portable SPR Sensor

**Figure 1:**
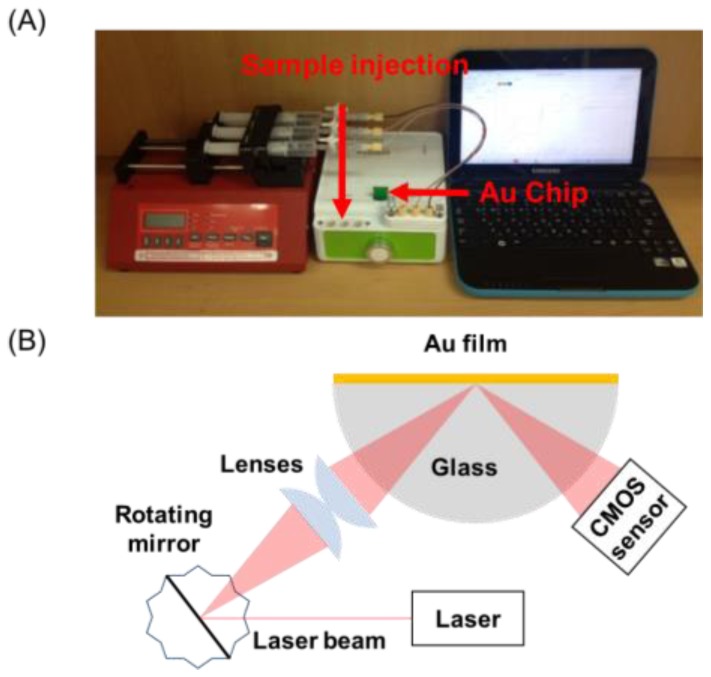
(**A**) Photograph of the portable surface plasmon resonance (SPR) system combined with a syringe pump and laptop. The Au chip was installed in the portable SPR system, and the sample solution was injected with the syringe pump. The SPR response was displayed on the laptop. (**B**) Schematic illustration of the portable SPR system based on the modulation of laser light by a rotating mirror.

Figure 1A shows the palm-sized SPR system (middle), combined with a syringe pump (left) and laptop (right). The Au chip was installed in the portable SPR system, and reagent solutions were injected by the syringe pump. The SPR response was displayed on the laptop. Figure 1B shows a schematic illustration of the portable SPR system based on the modulation of laser light by a rotating mirror. Conventional SPR systems adopt the prism-based Kretschmann configuration, in which a laser is used as the light source and the SPR angle is measured [24]. Oscillation mirror-based instruments, in which a mirror adjusts the incident angle at which surface plasmons are excited, have also been widely used [24]. These SPR systems, however, are unsuitable for portable applications. For the construction of the hand-held SPR device, we integrated a rotating mirror instead of the prism into the SPR system. The incident laser was modulated with a rotating mirror, where part of the reflected and diverged light from the center of the rotating mirror was focused on the Au surface. The reflected light was detected with a complementary metal-oxide-semiconductor sensor. This portable SPR system can eliminate the deterioration in image quality of the reflected laser light, originating from the coherency of the laser source. The SPR signal was transferred to a laptop via USB port, and the flow cell consisted of three channels with dimensions of 5.5 mm (L) × 1.0 mm (W) × 0.2 mm (D) on a single SPR chip.

**Figure 2:**
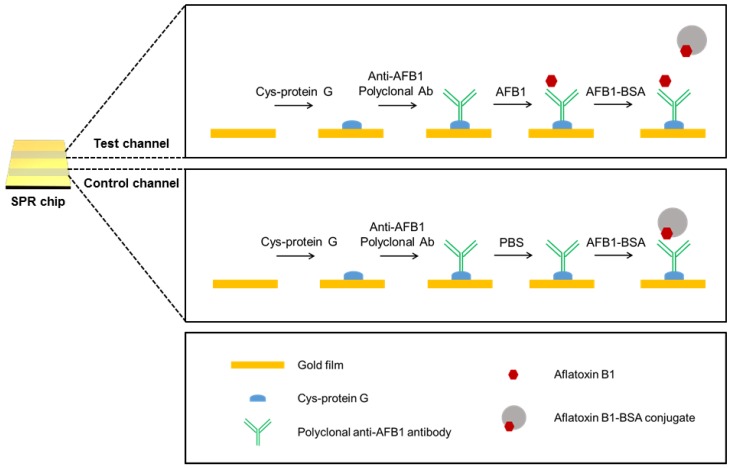
Schematic illustration of aflatoxin B1 (AFB1) detection using the portable SPR sensor.

The portable SPR device-based AFB1 detection procedure is illustrated in Figure 2. In this experiment, we adopted a competitive immunoassay to detect AFB1 sensitively. Because the SPR response is related to the mass change on the Au chip [23], molecules with low molecular weight, such as mycotoxins, are often difficult to detect with the SPR sensor. Therefore, additional SPR signal amplification steps or competitive binding assay have been employed to detect small molecules [25,26]. For the detection of AFB1, we first installed a pre-cleaned Au chip in the palm-sized SPR system, and the running buffer (PBS) and 0.1 mg/mL cysteine-protein G were flowed through both the test and control channel. Cysteine-protein G can bind to the Au chip through Au–S bonding [27,28]. Second, 0.1 mg/mL polyclonal anti-AFB1 antibody was immobilized on the Au chip through the binding of protein G to the Fc region of the antibody. The protein G-antibody retains the optimal conformation for interaction with AFB1, enabling efficient detection compared with a randomly immobilized antibody. Third, the AFB1 sample solution was flowed through the test channel, and the PBS solution was flowed through the control channel. Lastly, 1 mg/mL AFB1-BSA conjugate and PBS solution were injected into both channels. By comparing the SPR responses of the test and control channels, we could detect AFB1. When the sample solution contains AFB1, AFB1 binds to the Au chip, and AFB1-BSA flows without binding. Because the molecular weight of AFB1 is not large enough to produce an SPR signal, a weak SPR signal is obtained in the test channel. When the sample solution contains no AFB1, AFB1-BSA binds to the Au chip. The binding of heavy AFB1-BSA (66,775 Da) produces a large change in the SPR response. Since we flowed only buffer solution through the control channel, the SPR response increased to its maximum in the control channel. Consequently, we could quantitatively detect AFB1 through comparison of the SPR responses in the test and control channels.

### 3.2. Quantitative and Selective Detection of AFB1

**Figure 3:**
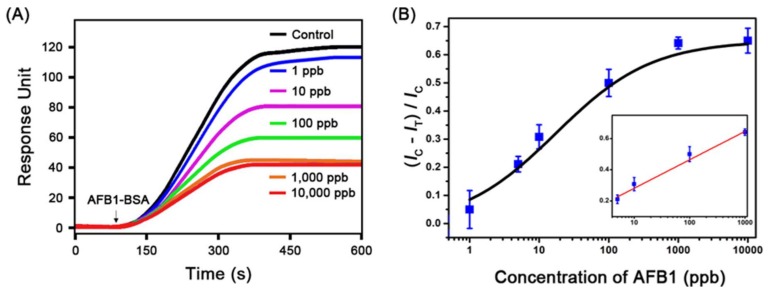
(**A**) SPR response curves obtained in the control channel (black line) and test channels by varying the AFB1 concentration (1, 10, 100, 1000, and 10,000 ppb). The SPR response intensity decreased with increasing AFB1 concentration because the amount of bound AFB1-BSA is inversely proportional to the AFB1 concentration. (**B**) Plot of (*I*_C_ − *I*_T_)/*I*_C_ versus the concentration of AFB1. *I*_C_ and *I*_T_ represent the mean SPR response intensity in the control and test channels, respectively. The red linearly fitted line. The data represent the mean plus standard deviation from three measurements.

To evaluate the sensing performance of the present portable SPR-based method in response to AFB1, we prepared AFB1 standard sample solutions at various concentrations from 1 to 10,000 ppb and tested the solutions, as depicted in Figure 2. The resultant SPR response curves are shown in Figure 3A. In the control channel, the highest response intensity was observed (black line of Figure 3A) after the injection of the AFB1-BSA conjugate. In the test channels, the SPR response intensity increased as the concentration of AFB1 was reduced from 10,000 to 1 ppb, as the amount of bound AFB1-BSA was inversely proportional to the AFB1 concentration. Figure 3B shows the plot of (*I*_C_ − *I*_T_)/*I*_C_ versus the AFB1 concentration. *I*_C_ and *I*_T_ represent the mean SPR response intensity in the control and test channels, respectively. The plot verifies that the quantitative detection of AFB1 is feasible. The fitted line was determined to be *y* = 0.18265*x* + 0.09848 with an *R*^2^ value of 0.98831. We estimate that the limit of detection (LOD) is 2.51 ppb, that the limit of quantification (LOQ) is 16.32 ppb, and that the quantitative dynamic range is 16~200 ppb (*x* = log concentration of AFB1). Previously reported SPR-based AFB1 sensing methods have shown a detection limit of 3 ppb using large BIAcore SPR equipment [29,30]. These methods, however, required additional AFB1 or Au chip surface modification steps for immobilizing AFB1 or AFB1-BSA conjugates on the sensor chip before flowing the mixture of AFB1 and anti-AFB1 antibody [29,30]. Here, we suggest a simpler way of identifying AFB1 without the abovementioned inconvenient steps, achieving sufficient results for satisfying regulatory limits.

**Figure 4:**
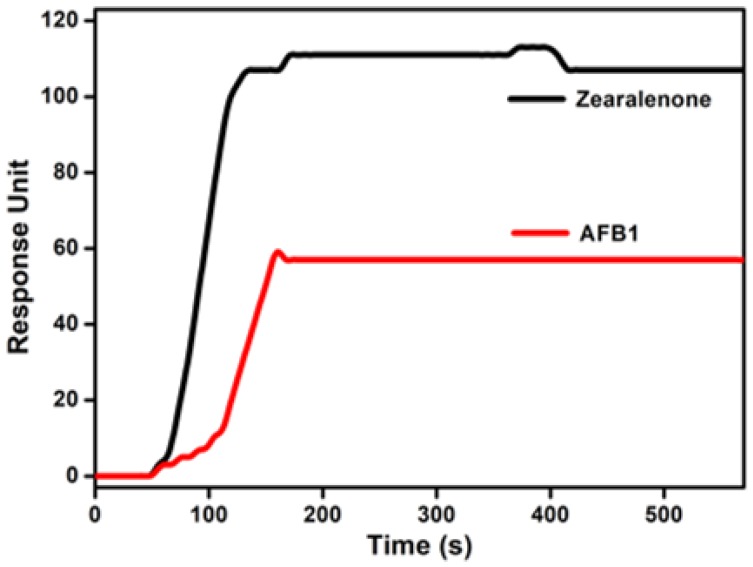
SPR response curves obtained in test channels after the detection of AFB1 and zearalenone, respectively. The concentration of both mycotoxins is 100 ppb. The SPR response was higher in the presence of zearalenone than in the presence of AFB1, indicating the selective detection of AFB1.

We also examined the selectivity of the portable SPR-based AFB1 sensing method. For the selectivity test, a 100 ppb zearalenone solution was prepared and flowed into the test channel of the SPR system. Zearalenone is an F-2 mycotoxin and a potent estrogenic metabolite produced by some *Fusarium* and *Gibberella* species [31]. Zearalenone can cause infertility, abortion, or other reproductive problems [32]. Figure 4 shows the SPR response curves obtained from two test channels with zearalenone (black line) and AFB1 (red line), respectively. A strong SPR response was obtained after the flow of zearalenone and AFB1-BSA. Since zearalenone could not bind to the chip, AFB1-BSA could successfully bind to the antibody, resulting in a large SPR response. On the other hand, a weak SPR response was measured after the flow of AFB1 (100 ppb) and AFB1-BSA. Because previously injected target AFB1 had already bound to the chip, it was difficult for AFB1-BSA to bind with the antibody. This result suggests that the present method can detect AFB1 selectively without a cross reaction of zearalenone.

### 3.3. The On-Site Detection of AFB1 in Grains

**Figure 5:**
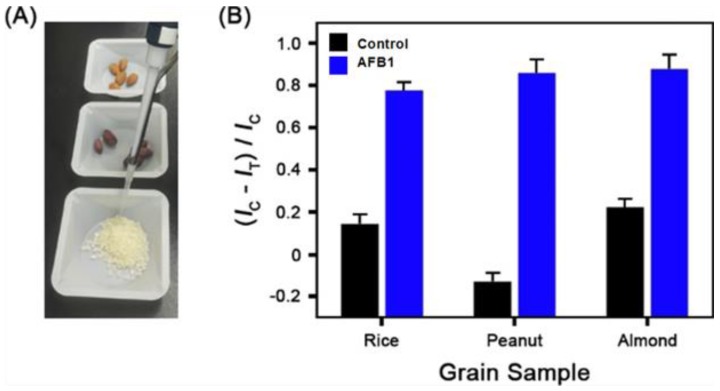
(**A**) Photograph of the AFB1-spiked rice, peanut, and almond samples. (**B**) Plot of (*I*_C_ − *I*_T_)/*I*_C_ versus the grain sample. The blue bars were obtained from the AFB1-spiked grain samples, and the black bars from pure grain samples. *I*_C_ and *I*_T_ represent the mean SPR response intensity in the control and test channels, respectively.

Finally, we tried to detect AFB1 in food samples using the portable SPR device. Rice, peanut, and almond were used as food matrices because they are the foods most commonly polluted by AFB1 [1]. Figure 5a shows a photograph of the AFB1-spiked rice, peanut, and almond samples. Five grams of these samples were spiked with 100 ppb AFB1. For the detection of AFB1, each spiked sample was incubated in 25 mL of methanol/water (70:30, v/v), and this mixture was shaken for 10 min. Next, the mixture was filtered with a 0.45 μm syringe filter, and the extract was used for analysis. Figure 5b shows the plot of (*I*_C_ − *I*_T_)/*I*_C_ versus the grain sample. When AFB1 was spiked in the grain samples, the (*I*_C_ − *I*_T_)/*I*_C_ value increased (blue bar). When the grain samples had no AFB1, the value decreased (black bar). This result clearly demonstrates the AFB1 sensing capability of the present method in grain samples.

## 4. Conclusions

We developed a miniaturized SPR system for the sensitive and on-site detection of AFB1 in grains. For AFB1 detection, the antibody was immobilized on the SPR Au chip through cysteine-tagged protein G, and the AFB1-BSA conjugate was flowed onto the chip after AFB1 sample treatment. The SPR response was induced by the AFB1-BSA conjugate, as it is relatively heavier than AFB1, and the competitive reaction between the AFB1-BSA conjugate and AFB1 induced an inverse relationship between the SPR response intensity and the AFB1 concentration. By using this portable SPR-based competitive immunoassay, the sensor achieved a low LOD (2.51 ppb), LOQ (16.32 ppb), and AFB1 recovered from spiked food samples was successfully detected. In addition, the result showed high selectivity against another mycotoxin. However, the proposed sensing method still has limitations in terms of reuse of the sensor, quantitative analysis in food samples, and costs of fabrication. If further research on the sophisticated fabrication and effective surface modification of the chips is carried out, we expect that this portable sensor will be useful for analyzing a variety of hazardous molecules in real time and on site.

## References

[1] Rustom I.Y.S. (1997). Aflatoxin in food and feed: Occurrence, legislation and inactivation by physical methods. Food Chem..

[2] Rajeev B., Ravishankar V.R., Karim A.A. (2010). Mycotoxins in Food and Feed: Present Status and Future Concerns. Compr. Rev. Food Sci. Food Saf..

[3] Hamid A.S., Tesfamariam I.G., Zhang Y., Zhang Z.G. (2013). Aflatoxin B1-induced hepatocellular carcinoma in developing countries: Geographical distribution, mechanism of action and prevention. Oncol. Lett..

[4] Lei J., Li P., Zhang Q., Wang Y., Zhang Z., Ding X., Zang W. (2014). Anti-idiotypic nanobody-phage based real-time immuno-PCR for detection of hepatocarcinogen aflatoxin in grains and feedstuffs. Anal. Chem..

[5] Milicevic D.R., Skrinjar M., Baltic T. (2010). Real and perceived risks for mycotoxin contamination in foods and feeds: Challenges for food safety control. Toxins.

[6] Medina A., Rodriguez A., Magan N. (2014). Effect of climate change on Aspergillus flavus and aflatoxin B1 production. Front. Microbiol..

[7] Wu F., Guclu H. (2012). Aflatoxin regulations in a network of global maize trade. PLoS ONE.

[8] Liu B.-H., Hsu Y.-T., Lu C.-C., Yu F.-Y. (2013). Detecting aflatoxin B1 in foods and feeds by using sensitive rapid enzyme-linked immunosorbent assay and gold nanoparticle immunochromatographic strip. Food Control.

[9] Zheng M.Z., Richard J.L., Binder J. (2006). A review of rapid methods for the analysis of mycotoxins. Mycopathologia.

[10] Wejdan S.K., Bahruddin S., Chew B.Y., Nor H.H., Abdussalam S.M.A., Muhammad I.S., Salleh B. (2010). Determination of aflatoxins in animal feeds by HPLC with multifunctional column clean-up. Food Chem..

[11] Reddy T.V., Viswanathan L., Venkitasubramanian T.A. (1970). Thin-layer chromatography of aflatoxins. Anal. Biochem..

[12] Otta K.H., Papp E., Bagocsi B. (2000). Determination of aflatoxins in food by overpressured-layer chromatography. J. Chromatogr. A.

[13] Lee N.A., Wang S., Allan R.D., Kennedy I.R. (2004). A rapid aflatoxin B1 ELISA: Development and validation with reduced matrix effects for peanuts, corn, pistachio, and Soybeans. J. Agric. Food Chem..

[14] Spinella K., Mosiello L., Palleschi G., Vitali F. (2013). Development of a QCM (Quartz Crystal Microbalance) Biosensor to the Detection of Aflatoxin B1. OJAB.

[15] Adanyi N., Levkovets I.A., Rodriguez-Gil S., Ronald A., Varadi M., Szendro I. (2007). Development of immunosensor based on OWLS technique for determining Aflatoxin B1 and Ochratoxin A. Biosens. Bioelectron..

[16] Zhou L., Li R., Li Z., Xia Q., Fang Y., Liu J. (2012). An immunosensor for ultrasensitive detection of aflatoxin B1 with an enhanced electrochemical performance based on graphene/conducting polymer/gold nanoparticles/the ionic liquid composite film on modified gold electrode with electrodeposition. Sens. Actuators B Chem..

[17] Lily A.M., Wei S., Xin X., Yan Z., Meng Y., Ivan R.K., Wang S. (2013). Colloidal gold based immunochromatographic strip for the simple and sensitive determination of aflatoxin B1 and B2 in corn and rice. Microchim. Acta.

[18] Homola J. (2003). Present and future of surface plasmon resonance biosensors. Anal. Bioanal. Chem..

[19] Nguyen H.H., Park J., Kang S., Kim M. (2015). Surface plasmon resonance: A versatile technique for biosensor applications. Sensors.

[20] Kong M., Sim J., Kang T., Nguyen H.H., Park H.K., Chung B.H., Ryu S. (2015). A novel and highly specific phage endolysin cell wall binding domain for detection of Bacillus cereus. Eur. Biophys. J..

[21] Shin Y.-B., Kim H.M., Jung Y., Chung B.H. (2010). A new palm-sized surface plasmon resonance (SPR) biosensor based on modulation of a light source by a rotating mirror. Sens. Actuator B Chem..

[22] Homola J. (2008). Surface plasmon resonance sensors for detection of chemical and biological species. Chem. Rev..

[23] Patching S.G. (2014). Surface plasmon resonance spectroscopy for characterisation of membrane protein-ligand interactions and its potential for drug discovery. Biochim. Biophys. Acta.

[24] Nguyen H.H., Yi S.Y., Woubit A., Kim M. (2016). A Portable Surface Plasmon Resonance Biosensor for Rapid Detection of Salmonella typhimurium. Appl. Sci. Converg. Technol..

[25] Hodnik V., Anderluh G. (2009). Toxin detection by surface plasmon resonance. Sensors.

[26] Matsui J., Akamatsu K., Hara N., Miyoshi D., Nawafune H., Tamaki K., Sugimoto N. (2005). SPR sensor chip for detection of small molecules using molecularly imprinted polymer with embedded gold nanoparticles. Anal. Chem..

[27] Lee J.M., Park H.K., Jung Y., Kim J.K., Jung S.O., Chung B.H. (2007). Direct immobilization of protein g variants with various numbers of cysteine residues on a gold surface. Anal. Chem..

[28] Kim H., Kang D.Y., Goh H.J., Oh B.K., Singh R.P., Oh S.M., Choi J.W. (2008). Analysis of direct immobilized recombinant protein G on a gold surface. Ultramicroscopy.

[29] Van D.G.B., Spath S., Dietrich H., Stigter E., Boonzaaijer G., Van Osenbruggen T., Koopal K. (2003). Biosensors and multiple mycotoxin analysis. Food Control.

[30] Daly S.J., Keating G.J., Dillon P.P., Manning B.M., O’Kennedy R., Lee H.A., Morgan M.R. (2000). Development of surface plasmon resonance-based immunoassay for aflatoxin B(1). J. Agric. Food Chem..

[31] Zinedine A., Soriano J.M., Molto J.C., Manes J. (2007). Review on the toxicity, occurrence, metabolism, detoxification, regulations and intake of zearalenone: An oestrogenic mycotoxin. Food Chem. Toxicol..

[32] Gromadzka K., Waskiewicz A., Chełkowski J., Golinski P. (2008). Zearalenone and its metabolites: Occurrence, detection, toxicity and guidelines. World Mycotoxin J..

